# Glepaglutide, a novel glucagon-like peptide-2 agonist, has anti-inflammatory and mucosal regenerative effects in an experimental model of inflammatory bowel disease in rats

**DOI:** 10.1186/s12876-023-02716-4

**Published:** 2023-03-21

**Authors:** Jolanta Skarbaliene, Jesper Mosolff Mathiesen, Bjarne Due Larsen, Christian Thorkildsen, Yvette Miata Petersen

**Affiliations:** 1 Research and Development, Zealand Pharma A/S, Sydmarken 11, 2860 Søborg, Denmark; 2 Present Address: Pharvaris GmbH, 6300, Grafenauweg 8, Zug, Switzerland; 3 Present Address: Hoffmann-La Roche, Grenzacherstrasse 124, 4070 Basel, Switzerland

**Keywords:** Glepaglutide, GLP-2, Anti-inflammatory, Mucosal healing, Inflammatory bowel disease

## Abstract

**Background:**

Glucagon-like peptide-2 (GLP-2) enhances intestinal repair and attenuates inflammation in preclinical inflammatory bowel disease (IBD) models, making GLP-2 analogues attractive candidates for IBD therapy. Glepaglutide is a long-acting GLP-2 receptor agonist in clinical development for treatment of short bowel syndrome. Here, we investigated if glepaglutide is therapeutically beneficial in rats with small intestinal inflammation.

**Methods:**

Small intestinal inflammation was induced with indomethacin in naive Wistar rats, followed by glepaglutide administration at different disease stages. Glepaglutide was administered in co-treatment and post-treatment regimens. Small intestinal length and concentrations of inflammatory markers α-1-acid glycoprotein and myeloperoxidase were used to assess anti-inflammatory effects. Small intestinal mass was evaluated to determine intestinotrophic effects.

**Results:**

Glepaglutide co- and post-treatment significantly reduced severity of small intestinal inflammation, evidenced by reversed small intestinal shortening and decreased α-1-acid glycoprotein and/or myeloperoxidase concentration(s). Co- and post-treatment with glepaglutide also significantly increased small intestinal mass, indicating intestinal regenerative effects. Similar effects were observed in naive rats after glepaglutide treatment.

**Conclusion:**

Glepaglutide has anti-inflammatory and intestinotrophic effects without the need for pre-treatment in a rat model of small intestinal inflammation. Thus, glepaglutide is of potential clinical interest for patients with IBD.

**Supplementary Information:**

The online version contains supplementary material available at 10.1186/s12876-023-02716-4.

## Background

Inflammatory bowel disease (IBD) is characterized by non-infectious chronic inflammation of the gastrointestinal tract. It primarily includes Crohn’s disease (CD), which can affect any segment of the gastrointestinal tract from the mouth to the anus, and ulcerative colitis (UC), which is limited to the colonic mucosa. The cause of IBD is unknown. It is considered to be the result of an inappropriate immune response against environmental factors, including luminal and microbial antigens, in genetically susceptible hosts [1]. In 2017, nearly 3.9 million female patients and nearly 3.0 million male patients were living with IBD worldwide. The prevalence of IBD increased substantially in many regions from 1990 to 2017, which poses substantial social and economic burdens on health systems [2].

Conventional first-line basic therapies (aminosalicylates, antibiotics, and glucocorticosteroids) have traditionally focused on treating inflammation and inducing remission. Their benefits are often short-lived and many patients do not respond effectively to these treatments [3–5]. In the event of unresponsiveness to conventional treatments, targeted biologics may be administered [3]. They have markedly enhanced clinical outcomes, most likely owing, in part, to the healing effects these compounds have demonstrated on the intestinal mucosa [3, 6–8]. However, owing to the potentially serious side effects linked to these biologics [8, 9], clinical use is mainly recommended in patients who are incomplete responders or intolerant to conventional drugs [8]. Conventional and biologic therapies typically target the immune response, but evidence for robust improvement of intestinal barrier function is scarce.

To date, the majority of IBD therapies have focused on downregulating intestinal inflammation by manipulating the immune system. This has led to great advancements in the medical treatment of a disease that has only had surgical treatment options in the past. Despite these therapeutic advances, many patients with IBD still require surgery [10]. Patients with CD are especially at risk of developing short bowel syndrome following recurrent intestinal resections.

Although multiple effective therapeutic options exist for the treatment of IBD, a proportion of patients will either not respond or lose response to therapy. This emphasizes the need for new therapeutic options in patients with IBD that allow for the long-term treatment of gastrointestinal diseases without impairing the immune system. In addition, recent work has highlighted the importance of the intestinal microbiome and mucosal barrier function in disease pathophysiology [10].

Glucagon-like peptide-2 (GLP-2) is a 33-amino-acid intestinal peptide released from intestinal L-cells that exerts its function through the GLP-2 receptor (GLP-2R). It is expressed predominantly in the intestinal tract [11, 12]. Studies conducted over the past two decades have identified GLP-2 as an important regulator of intestinal growth and function [13, 14].

Previously, significant focus was placed on the mucosal growth-promoting effects of GLP-2, mediated via stimulation of crypt cell proliferation and inhibition of villus cell apoptosis [15, 16]. Subsequently, GLP-2 was increasingly recognized as a potent anti-inflammatory peptide that acts directly to improve inflammation status in experimental models of intestinal inflammation [17–20]. The potent anti-inflammatory role for GLP-2 in IBD models is well documented in the literature [17–20]. Sigalet et al. [17], L’Heureux and Brubaker [21], and Boushey et al. [19] showed that GLP-2 had clear anti-inflammatory effects in mice with chemically induced colitis and indomethacin (INDO)-induced small intestinal inflammation. Alavi et al. demonstrated that continuous GLP-2 infusion had anti-inflammatory effects in a rat model of spontaneously developing small intestinal inflammation [20]. In a study with another GLP-2 agonist, [Gly^2^]GLP-2 (teduglutide), Boushey et al. showed that pre-treatment was necessary to attenuate inflammation [19].

The anti-inflammatory effects are thought to act via multiple mechanisms. The transcription factor nuclear factor κ-ligand B (NFκB) controls the production and secretion of various pro-inflammatory cytokines and chemokines that play a role in IBD pathophysiology. A growing body of research indicates that GLP-2R agonists suppress NFκB activity [22]. GLP-2 also improves intestinal barrier function in both healthy conditions and disease models [23–25]. Taken together, GLP-2 analogues may have the potential to become a new therapeutic option in IBD, a condition characterized by destruction of the gastrointestinal epithelium.

Glepaglutide is a long-acting GLP-2R agonist developed by Zealand Pharma and is currently in clinical development for the treatment of short bowel syndrome [26, 27]. The objective of this study was to investigate whether glepaglutide was therapeutically beneficial in rats with small intestinal inflammation.

## Methods

### Glepaglutide and INDO

Glepaglutide, formerly called ZP1848, was synthesized batchwise by means of a standard solid-phase procedure using the Fmoc strategy. The crude material was purified using preparative high-performance liquid chromatography. The identity of the pure material was confirmed by electrospray mass spectrometry [28]. Immediately before use, glepaglutide was dissolved in vehicle (15 mM histidine and 271 mM mannitol) and administered subcutaneously (SC) at a dose of 5 ml/kg. INDO (Sigma-Aldrich, Denmark, product number I7378-5G; 50 mg) was freshly dissolved in 14.3 ml of 0.9% NaCl supplemented with 27.1 mg NaH_2_PO_4_, H_2_O, and 12 mg NaOH. Prior to administration at a dose of 2 ml/kg, the pH was adjusted to pH 6.8–7.3 with HCl.

### Animals

Naive Wistar rats (Taconic, Ll. Skensved, Denmark), weighing 170–230 g, were used in the experiments. The animals were allowed at least 7 days of acclimatization in Makrolon type III cages before study start. Animals were housed (two per cage) under a 12:12-h light/dark cycle. Food (standard Altromin type 1321, Brogården, Denmark) and drinking water (domestic quality tap water with added citric acid to approximately pH 3) were provided ad libitum.

### Glepaglutide intestinotrophic effect in naive rats

**Fig. 1:**
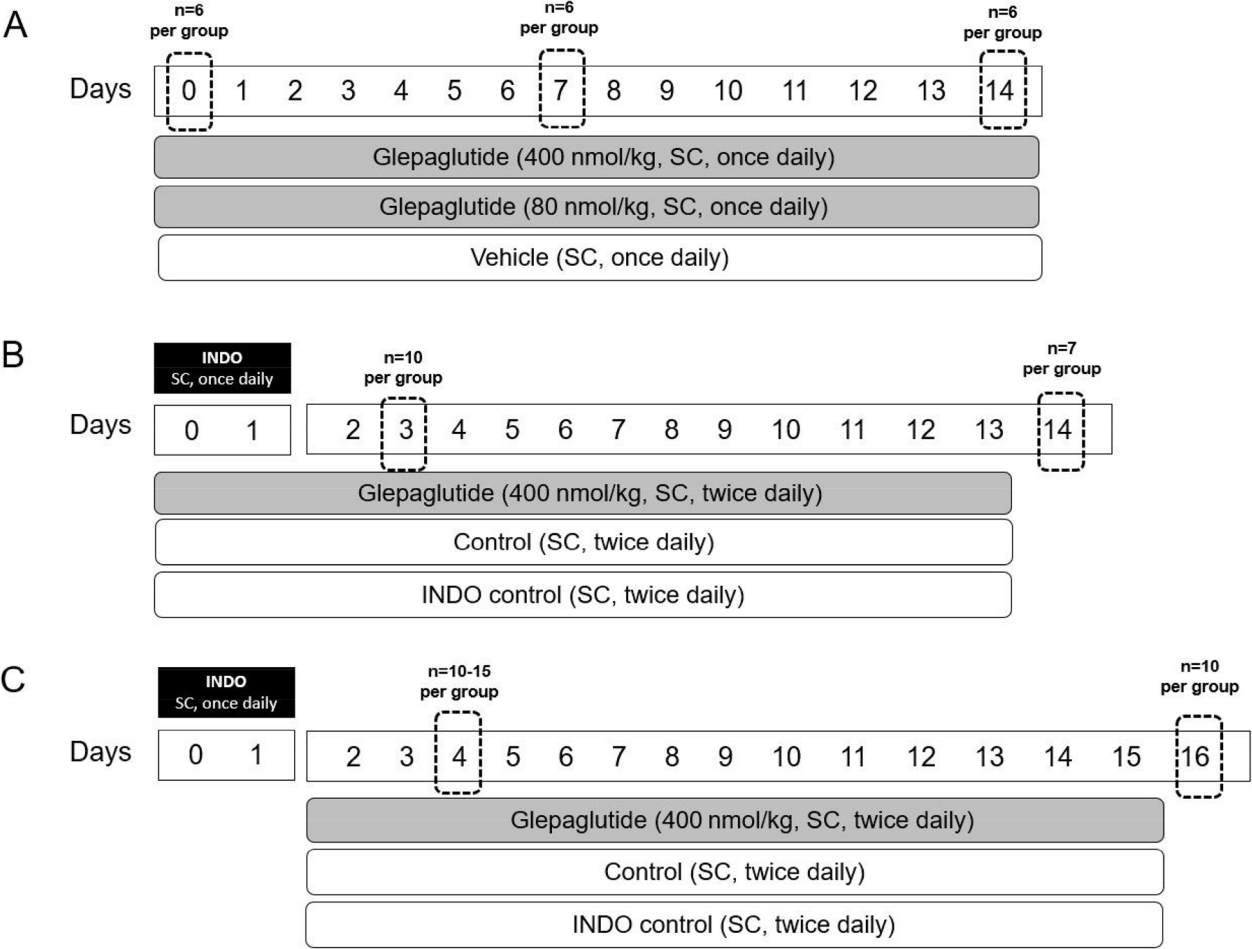
Study design diagram for the assessment of the intestinotrophic effect of glepaglutide in naive rats and its effects in the INDO-induced small intestinal inflammation model in a co-treatment and post-treatment regimen. **A** Naive Wistar rats were treated with glepaglutide 400 nmol/kg, glepaglutide 80 nmol/kg, or vehicle (SC, twice daily) for 14 days. **B/C** Naive Wistar rats were treated with INDO 7 mg/kg (SC, once daily) for 2 consecutive days. Rats with small intestinal inflammation were treated with glepaglutide 400 nmol/kg, control, or INDO control (SC, twice daily) in **B** co-treatment for 14 days or **C** post-treatment for 16 days. The dashed rectangles show sacrifice dates. *INDO*, indomethacin; *SC*, subcutaneously

Glepaglutide (80 nmol/kg or 400 nmol/kg, SC, once daily) or vehicle (SC, once daily) were administered for 14 days to naive Wistar rats to assess the intestinotrophic properties of the peptide, before initiating studies in the small intestinal inflammation model (Fig. 1A). Overall, 18 rats were included per arm, with 6 rats each sacrificed on day 0, day 7, and day 14.

### Glepaglutide effects in the INDO-induced small intestinal inflammation model

Small intestinal inflammation was induced by administration of INDO (7 mg/kg, SC, once daily) on 2 days consecutively (day 0 and day 1) in male Wistar rats (Fig. 1B and C). Two treatment regimens were studied: glepaglutide (400 nmol/kg, SC, twice daily) was administered in a co-treatment regimen (Fig. 1B – administered at inflammation onset and throughout), and a post-treatment regimen (Fig. 1C – administered after inflammation onset and throughout).

This is a well-established model of CD, because the small intestinal inflammation induced by INDO shares a number of pathological similarities with CD [29]. INDO produces small intestinal inflammation in a dose-dependent manner [29, 30]. Yamada et al. showed that one injection SC of INDO at 7.5 mg/kg caused acute damage to the intestine with mucosal erosions and ulcerations in the distal jejunum and proximal ileum. Inflammation was localized primarily on the mesenteric side of the mid-small intestine and persisted throughout the first 3 days, then gradually resolved within 1 week [29]. Two injections SC of INDO 7.5 mg/kg 24 h apart resulted in more severe chronic intestinal inflammation with multiple mucosal ulcers on the mesenteric side of the small intestine in the injected rats [29, 31]. With this regimen, the INDO-induced chronic damage was more pronounced in the region of the mid-small intestine and the inflammation persisted for approximately 2 weeks [29]. In this study, a dose of INDO 7 mg/kg was used based on the results of an INDO-induced small intestinal inflammation model validation study conducted by Zealand Pharma. The study showed that administration of INDO at 7 mg/kg (SC, once daily) on 2 days consecutively (day 0 and day 1) in male Wistar rats was sufficient and appropriate to introduce macroscopic features of inflammation (adhesions, intestinal wall thickening and ulcerations [32]), while 3 mg/kg was insufficient for inducing inflammation and 15 mg/kg led to exaggerated mortality (data not shown).

Two groups of controls, INDO controls (administered with INDO and treated with vehicle) and controls (administered with saline solution and treated with vehicle), were included in the studies (Fig. 1B and C). Groups of rats (n = 7–15) were sacrificed during the active inflammation phase (2–3 days after inflammation onset), on day 3 for the co-treatment regimen (Fig. 1B) and on day 4 for the post-treatment regimen (Fig. 1C), to determine small intestinal length, jejunal and ileal mass, and α-1-acid glycoprotein (α-1-AGP) and myeloperoxidase (MPO) concentrations in the jejunum and ileum. Equivalent groups of rats were sacrificed during the body weight recovery phase (14–15 days after inflammation onset), on day 14 for the co-treatment regimen (Fig. 1B), and on day 16 for the post-treatment regimen (Fig. 1C), to determine jejunal and ileal mass. Body weight and survival were recorded daily.

Animals were anesthetized with carbon dioxide and then sacrificed by cervical dislocation. The small intestine (from the pylorus to the ileocecal junction) was dissected away from the abdomen and the surrounding mesenteric fat. Small intestinal length was measured after weighing the small intestine with a 1 g weight to obtain uniform tension. Thereafter, the small intestine was divided into two segments of equal length labeled as the jejunum and ileum. Segments were gently flushed with 0.9% saline, padded dry, and weighed. A 5 cm biopsy was collected 10 cm distal from the start of the jejunal segment, and another 5 cm biopsy was collected 10 cm distal from the start of the ileal segment [29–31]. The biopsies were immediately frozen in liquid nitrogen and stored at -80 °C for later analysis of concentrations of α-1-AGP and MPO using commercially available enzyme-linked immunosorbent assay kits (Life Diagnostics [2510–2] and Hycult Biotech [HK 210], respectively). The preparation method was validated in house.

### In vitro activity of glepaglutide at the hGLP-2R

An in vitro cyclic adenosine monophosphate (cAMP) assay was conducted to assess the stimulation of cAMP accumulation by glepaglutide [33]. Human GLP-2 (hGLP-2) was used as a reference agonist. A cell line stably expressing hGLP-2 receptor (hGLP-2R) was generated by Lipofectamine (Invitrogen #18,324–012) transfection of human embryonic kidney (HEK) 293 cells (ATCC #CRL-1573) with a mammalian expression vector containing the complementary DNA encoding the hGLP-2R (natural variant dbSNP:rs17681684) and a G418 resistance gene. Following 4 weeks in culture in selection with 0.5 mg/ml G418, single clones were picked and tested in a functional GLP-2 receptor potency assay as described below. One clone was selected for use in compound profiling. For assaying, HEK293 cells stably expressing hGLP-2R were seeded at 30,000 cells per well in 96-well microtiter plates coated with 0.01% poly-L-lysine. Cells were grown overnight in 200 µl growth medium (DMEM with Glutamax-I [Invitrogen 61965], containing 10% v/v FBS [Invitrogen 10,270–106], 1% v/v PenStrep [Invitrogen 15140], 0.5 mg/ml Geneticin [Invitrogen 10,131–027], 1 mM sodium pyruvate [Invitrogen 11,360–039], and 1 × NEAA [Invitrogen 11140]). On the day of assaying, the growth medium was removed, and the cells were washed once with 150 μl Tyrode buffer (Tyrode’s salts, Sigma-Aldrich T2145) supplemented with 10 mM HEPES (Invitrogen 15,630), pH 7.4. To initiate cAMP accumulation, cells were incubated in 100 μl Tyrode buffer supplemented with 0.1% w/v alkali-treated casein (Sigma-Aldrich C4765) and 100 μΜ IBMX (Sigma-Aldrich I5879) and increasing concentrations of glepaglutide or hGLP-2 for 15 min at 37 °C. The reaction was stopped by decanting off the compound/buffer and replacing it with 80 µl lysis/detection buffer consisting of deionized water supplemented with 0.1% w/v BSA (Sigma-Aldrich A9430), 5 mM HEPES, and 0.3% v/v Tween-20 (Sigma-Aldrich P7949). After incubation at room temperature for 10 min, the cAMP content of 10 µl of the resulting cell lysate was estimated using the AlphaScreen® cAMP assay kit (Perkin Elmer 6760635 M) according to the manufacturer’s instructions.

Compound potency (EC_50_) and maximal effect (E_max_) values were estimated by computer-aided curve fitting using a three-parameter logistic non-linear model. This was done after normalizing the compound response relative to a saturating concentration of the reference agonist (hGLP-2) alone and the baseline cAMP level of the unstimulated receptor.

### Statistical analysis

Data are presented as mean ± standard error of the mean, unless otherwise stated. The intestinal growth-promoting effect of glepaglutide in naive rats was determined using a two-way analysis of variance (ANOVA, dose and time), and significant findings were further analyzed with the Bonferroni post hoc test. In the INDO model, a *t*-test was used to compare INDO control-treated rats with vehicle control-treated rats, and INDO control-treated rats with glepaglutide-treated rats. A *p* value of ≤ 0.05 was considered statistically significant. Grubbs’ test was used to test for outliers in the section, and outliers were not included in the analyses. Differences in survival between different groups were compared by the Cox–Mantel log-rank test.

## Results

### In vitro potency of glepaglutide at the hGLP-2R

The in vitro potency of glepaglutide was determined by its ability to stimulate cAMP formation in HEK293 cells recombinantly overexpressing hGLP-2R. Glepaglutide activated hGLP-2R with a similar potency to native hGLP-2, and the maximal agonist response of glepaglutide was likewise similar to that of hGLP-2 (Table S1).

### Glepaglutide intestinotrophic effect in naive rats

**Fig. 2:**
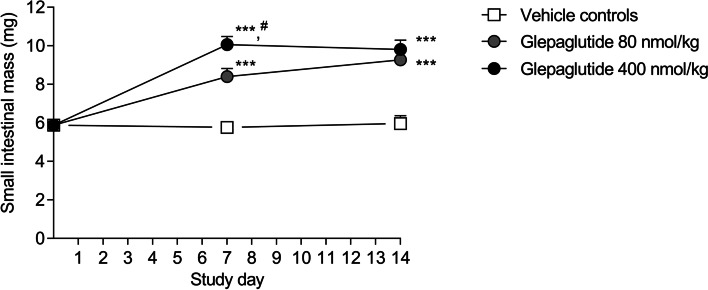
Small intestinal mass on day 7 and day 14 in naive Wistar rats administered glepaglutide. Glepaglutide was administered once daily (80 nmol/kg and 400 nmol/kg, SC). ***p < 0.001 vs vehicle controls, ^#^*p* < 0.05, 80 nmol/kg vs 400 nmol/kg glepaglutide in two-way ANOVA, followed by a Tukey multiple comparisons test. Data shown are mean ± SEM. *SC*, subcutaneously; *SEM*, standard error of the mean

Glepaglutide dose-dependently increased small intestinal mass in naive Wistar rats (Fig. 2), in line with observations made in a recent study in rats and dogs [34]. Small intestinal mass was dose-dependently increased after 7 days of daily dosing, with the 400 nmol/kg dose being significantly increased compared to the 80 nmol/kg dose (two-way ANOVA, Tukey multiple comparisons test, *p* < 0.05). In the 400 nmol/kg dose group, small intestinal mass rose to a plateau by day 7, and on day 14 small intestinal mass was similar in both dose groups (two-way ANOVA, Tukey multiple comparisons test, *p* = 0.5885). The 400 nmol/kg dose of glepaglutide was selected for use in the inflammation model because, at this dose, glepaglutide mediated a significant and sustained increase in small intestinal mass.

### Glepaglutide effects in the INDO-induced small intestinal inflammation model

Small intestinal inflammation induced by INDO 7 mg/kg was characterized by acute body weight loss (day 0 to day 4; *p* < 0.001), increased mortality (data not shown), decreased small intestinal length, increased jejunal and ileal mass, and increased α-1-AGP and MPO concentrations in the jejunum and ileum in the active inflammation phase. Body weight rose gradually after day 4 and was back to normal on day 14.

**Fig. 3:**
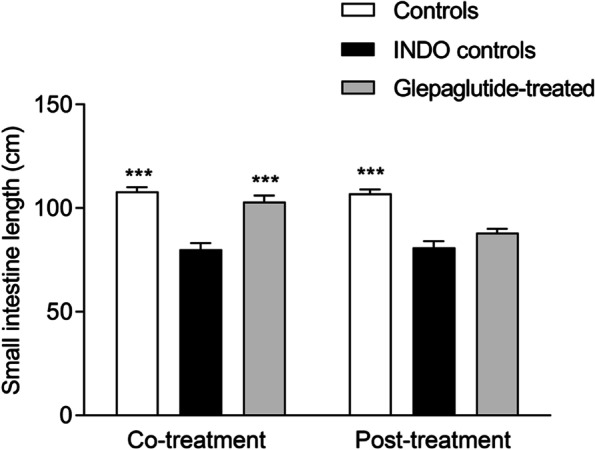
Effect of glepaglutide co-treatment or post-treatment on small intestinal length in the active inflammation phase. Effect of a co-treatment or post-treatment regimen of glepaglutide (400 nmol/kg, SC, twice daily) on small intestinal length (cm) was assessed in the active inflammation phase (2–3 days after inflammation onset), in Wistar rats with small intestinal inflammation. ***p < 0.001 vs INDO controls. Data shown are mean ± SEM. *INDO*, indomethacin; *SC*, subcutaneously; *SEM*, standard error of the mean

**Fig. 4:**
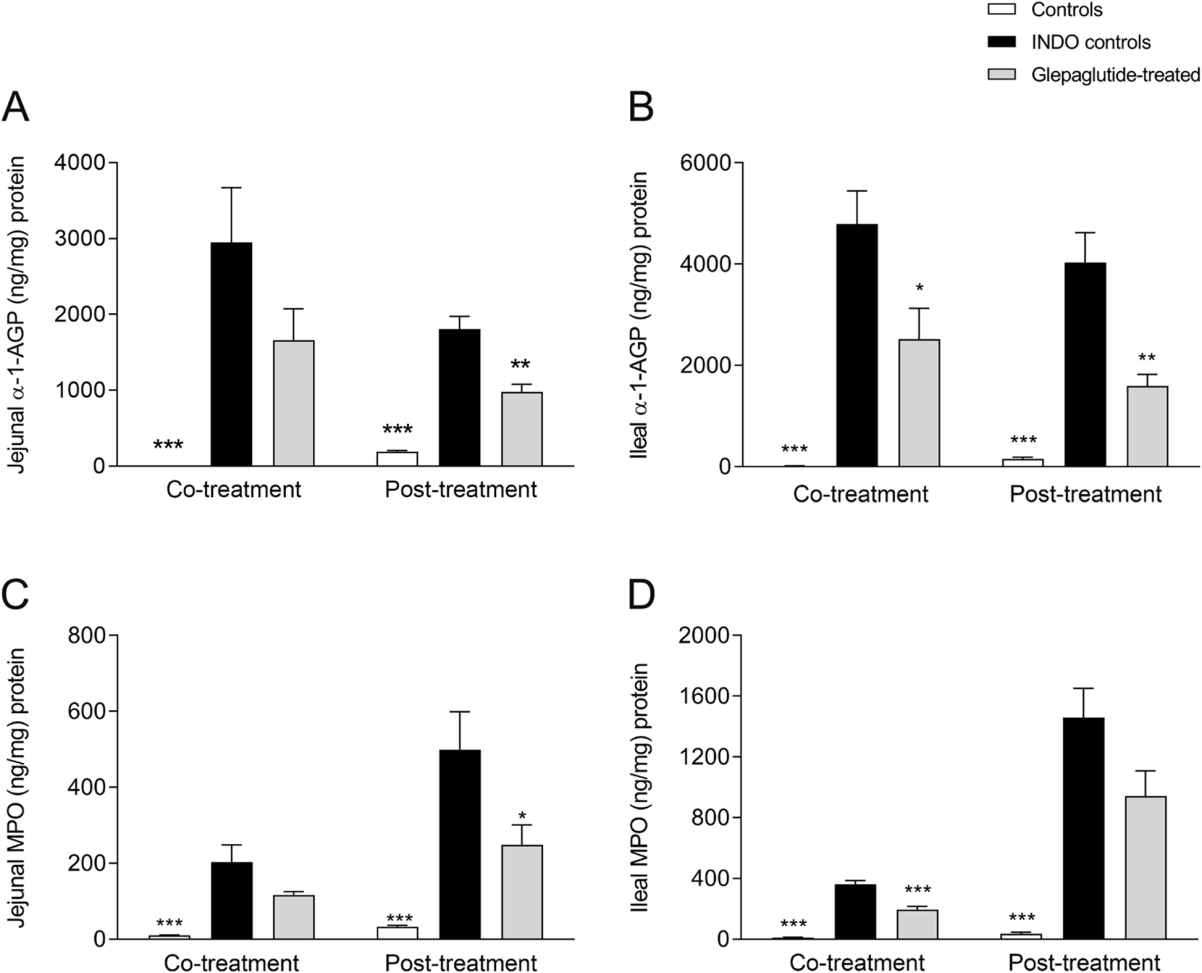
Effect of glepaglutide co-treatment or post-treatment on jejunal and ileal inflammatory markers during active inflammation. Effect of glepaglutide (400 nmol/kg, SC, twice daily) administered in a co-treatment or post-treatment regimen on **A** jejunal and **B** ileal α-1-AGP concentrations (ng/mg protein), and **C** jejunal and **D** ileal MPO concentrations (ng/mg protein), in the active inflammation phase (2–3 days after inflammation onset) in Wistar rats with small intestinal inflammation. *p ≤ 0.05, **p < 0.01, ***p < 0.001 vs INDO controls. Data shown are mean ± SEM. *α-1-AGP*, α-1-acid glycoprotein; *INDO*, indomethacin; *MPO*, myeloperoxidase; *SC*, subcutaneously; *SEM*, standard error of the mean

Co-treatment with glepaglutide significantly decreased acute body weight loss (*p* < 0.001; data not shown), reversed small intestinal shortening (*p* < 0.001; Fig. 3), and significantly decreased ileal α-1-AGP (*p* < 0.05; Fig. 4B) and ileal MPO (*p* < 0.001; Fig. 4D) concentrations. Post-treatment with glepaglutide had no effect on acute body weight loss (data not shown). However, it significantly reversed small intestinal shortening (*p* = 0.05; Fig. 3) and significantly decreased jejunal and ileal α-1-AGP (*p* < 0.01; Fig. 4A, B) and jejunal MPO (*p* < 0.05; Fig. 4C) concentrations in the active inflammation phase. Ileal mass was significantly increased in the glepaglutide co-treatment group during the active inflammation phase (*p* < 0.05; Fig. S2B). Both jejunal and ileal mass were significantly increased in the co- and post-treatment groups in the body weight recovery phase (Fig. S2A–D). Glepaglutide had no effect on survival in the regimens used.

## Discussion

IBDs are a heterogeneous group of conditions divided into two predominant groups, CD and UC. These conditions are characterized by chronic inflammation, a relapsing and remitting clinical course, requirement for lifelong medication, and often significant morbidity. Although multiple effective therapeutic options exist for the treatment of IBD, a proportion of patients will either not respond to or lose response to therapy. This emphasizes the significance of exploring and identifying therapies with novel therapeutic targets for patients with IBD [4].

GLP-2 is an intestinally derived hormone that enhances intestinal growth, digestion, absorption, barrier function, and blood flow in healthy animals. It also prevents damage and improves repair in preclinical models of enteritis and colitis, and following substantial small bowel resection. These beneficial effects of GLP-2 on the intestinal tract are largely recapitulated in humans with intestinal failure [14].

Glepaglutide is a long-acting GLP-2R agonist in clinical development for short bowel syndrome. Here, we have shown that glepaglutide activates the hGLP-2R in vitro with a potency similar to that of native GLP-2. In our study, glepaglutide also significantly increased small intestinal mass in naive Wistar rats. Based on the vast amount of published literature on the mechanism of action of GLP-2, we speculate that this growth effect of glepaglutide is mediated via stimulation of crypt cell proliferation and inhibition of villus cell apoptosis leading to increased mucosal growth [15, 16]. The intestinotrophic effects of glepaglutide were apparent 7 days after dosing started and were further increased (80 nmol/kg dose group) or maintained (400 nmol/kg dose group) after an additional week of dosing. This suggests that maximal small intestinal growth had been attained.

In addition, we have shown that glepaglutide administered at the time of the onset of inflammation or during the active inflammation phase was therapeutically active in a rat model of INDO-induced small intestinal inflammation. This model is well established as a model of CD, because the small intestine inflammation induced by INDO shares a number of pathological similarities with CD [29]. Both co- and post-treatment with glepaglutide (400 nmol/kg) had beneficial but slightly different effects on the studied parameters. Co-treatment with glepaglutide decreased acute body weight loss, reversed small intestinal shortening, and decreased ileal α-1-AGP and MPO concentrations. Post-treatment with glepaglutide had no effect on acute body weight loss, but reversed small intestinal shortening and decreased both jejunal and ileal α-1-AGP and jejunal MPO concentrations. The difference in the effect on body weight could be due to the difference in dosing regimens (3 days vs 2 days) during the active inflammation phase and the state of inflammation at the onset of treatment. Interestingly, post-treatment with glepaglutide had a more pronounced effect than co-treatment on jejunal and ileal α-1-AGP and jejunal MPO concentrations. This indicates that the intestine was responsive to treatment with glepaglutide despite the progressive inflammation-induced damage.

Our study further demonstrates that glepaglutide effectively decreases inflammation without the need for a pre-treatment period. This could be of great potential clinical interest in the IBD patient populations.

Inflammation within the small intestine increases epithelial cell proliferation as a means of repairing damage [35]. During the inflammation phase, small intestinal mass was significantly increased in INDO controls but decreased gradually as the animals recovered. The small intestinal mass was similar between glepaglutide-treated and INDO control animals during the active inflammation phase. Despite this, the significantly lower levels of α-1-AGP and MPO in the glepaglutide-treated animals suggest that the rise in small intestinal mass was due, at least in part, to other factors/mechanisms taking place in the inflamed intestine of the glepaglutide-treated animals. In both co- and post-treated animals, jejunal and ileal mass were markedly increased compared with INDO controls in the body weight recovery phase. We hypothesize that the significantly increased small intestinal mass in glepaglutide-treated animals was due to one or more of the following factors: increased cell proliferation, inhibition of apoptosis, increased blood flow to the small intestine, and/or increased protein synthesis [15, 16].

The anti-inflammatory and intestinal growth effects mediated by glepaglutide, coupled with the exclusive localization of the GLP-2R in the gastrointestinal tract, make this peptide an interesting therapeutic candidate for treatment of IBD. There is currently much focus on the importance of mucosal healing as a means of changing the natural course of disease in patients with IBD [6, 7]. Mucosal healing is expected to lead to symptom improvement, to decrease relapse and/or recurrence rates, to reduce complications such as the need for surgical interventions, and to abate the incidence of cancer and associated risk of death in IBD [6, 7]. Although the majority of conventional therapies, including glucocorticoids, with the exception of prednisolone, have little or no effecton mucosal healing [36], immunosuppressants and biologics are reported to heal the mucosa but have slow onsets of action, requiring several months of dosing before healing is achieved [7]. These insufficiencies of conventional therapies are thought to play a role in the inadequacies in IBD treatment [3, 7].

Use of GLP-2 agonists for the treatment of IBD is not a novel concept. The ability of teduglutide to induce remission or to reduce the Crohn’s disease activity index (CDAI) score by at least 100 points after 8 weeks of treatment was examined in patients with moderate to severe CD [37]. It was shown that teduglutide had a positive, dose-dependent effect on the CDAI score but that this failed to reach statistical significance. We speculate that the study was underpowered and that the assessment of mucosal healing, rather than the CDAI, would have been a better endpoint to assess the effect of treatment, because GLP-2 is known to act directly on the small intestinal mucosa. However, measurement of mucosal healing is still largely observational. It requires repeated invasive endoscopic examinations, sometimes with mucosal biopsies, and has only recently become more established [6].

## Conclusion

In summary, we have demonstrated that glepaglutide is a highly potent GLP-2R agonist with anti-inflammatory and mucosal regenerating effects in the rat model of INDO-induced small intestinal inflammation. Glepaglutide is therapeutically effective when administered at the onset of small intestinal inflammation, as well as during active inflammation. This suggests that this peptide could potentially be used in patients experiencing symptoms of an upcoming relapse or in those with an active relapse. In conclusion, our findings suggest that glepaglutide may be a therapeutic candidate with effects on mucosal healing and potential for long-term remission in IBD.

## Supplementary Information

**Additional file 1: Table S1.** Comparison of agonism of hGLP-2R (cAMP accumulation) by glepaglutide with human GLP-2. **Figure S2.** Effect of glepaglutide co-treatment and post-treatment on small intestinal mass in active inflammation and body weight recovery phase. **A** Co-treatment: Jejunum mass normalized to bodyweight (mg/kg) on day 3. **B** Co-treatment: Ileum mass normalized to bodyweight (mg/kg) on day 14. **C** Post-treatment: Jejunum mass normalized to bodyweight (mg/kg) on day 4. **D** Post-treatment: Ileum mass normalized to bodyweight (mg/kg) on day 16.

## Data Availability

The datasets used and/or analyzed during the current study are available from the corresponding author on reasonable request.

## Abbreviations

α-1-AGP: α-1-Acid glycoprotein
cAMP: Cyclic adenosine monophosphate
CD: Crohn’s disease
CDAI: Crohn’s disease activity index
EC_50_: Half maximal effective concentration
E_max_: Maximal effect
GLP-2: Glucagon-like peptide-2
GLP-2R: Glucagon-like peptide-2 receptor
HEK: Human embryonic kidney
hGLP-2: Human glucagon-like peptide-2
hGLP-2R: Human glucagon-like peptide-2 receptor
IBD: Inflammatory bowel disease
INDO: Indomethacin
MPO: Myeloperoxidase
NFκB: Nuclear factor κ-ligand B
SC: Subcutaneously
UC: Ulcerative colitis
