# Supplementary material for: Glepaglutide, a novel glucagon-like peptide-2 agonist, has anti-inflammatory and mucosal regenerative effects in an experimental model of inflammatory bowel disease in rats
Source: BMC Gastroenterol. 2023 Mar 21;23:79. doi: 10.1186/s12876-023-02716-4 (PMC10029296; doi:10.1186/s12876-023-02716-4)
Supplement: Supplementary file 1 — Additional file 1: Table S1. Comparison of agonism of hGLP-2R (cAMP accumulation) by glepaglutide with human GLP-2. Figure S2. Effect of glepaglutide co-treatment and post-treatment on small intestinal mass in active inflammation and body weight recovery phase. A Co-treatment: Jejunum mass normalized to bodyweight (mg/kg) on day 3. B Co-treatment: Ileum mass normalized to bodyweight (mg/kg) on day 14. C Post-treatment: Jejunum mass normalized to bodyweight (mg/kg) on day 4. D Post-treatment: Ileum mass normalized to bodyweight (mg/kg) on day 16. [file 12876_2023_2716_MOESM1_ESM.pdf]

Supplemental materials

Table S1. Comparison of agonism of hGLP-2R (cAMP accumulation) by glepaglutide with human GLP-2

	EC_{50} (nM)	pEC_{50} ± SEM	E_{max} ± SEM	n
Human GLP-2	0.025	10.60 ± 0.07	106 ± 3.7	5
Glepaglutide	0.093	10.03 ± 0.09	102 ± 3.7	5

cAMP, cyclic adenosine monophosphate; *EC_{50}*, half maximal effective concentration; *E_{max}*, maximal effect; *GLP-2*, glucagon-like peptide-2; *hGLP-2R*, human glucagon-like peptide-2 receptor; *pEC_{50}*, logarithmically transformed half maximal effective concentration ($pEC_{50} = -\log_{10}[EC_{50}]$); *SEM*, standard error of the mean

Figure S2. Effect of glepaglutide co-treatment and post-treatment on small intestinal mass in active inflammation and body weight recovery phase. **A** Co-treatment: Jejunum mass normalized to bodyweight (mg/kg) on day 3. **B** Co-treatment: Ileum mass normalized to bodyweight (mg/kg) on day 14. **C** Post-treatment: Jejunum mass normalized to bodyweight (mg/kg) on day 4. **D** Post-treatment: Ileum mass normalized to bodyweight (mg/kg) on day 16.

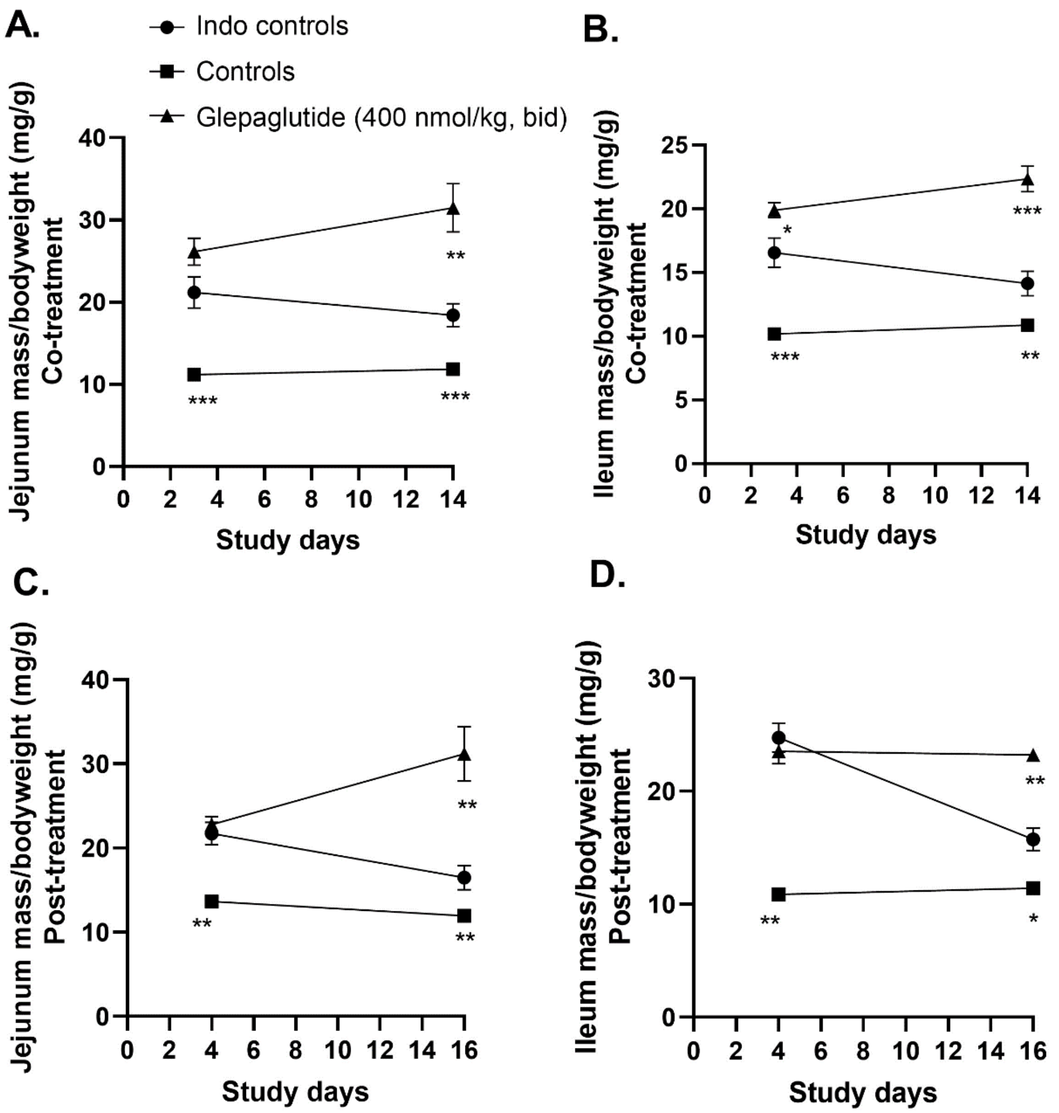

*$p < 0.05$, **$p < 0.01$, ***$p < 0.001$ vs INDO control group. Data shown are mean ± SEM

bid, twice a day, *INDO*, indomethacin; *SEM*, standard error of the mean
